# Colony, hanging drop, and methylcellulose three dimensional hypoxic growth optimization of renal cell carcinoma cell lines

**DOI:** 10.1007/s10616-016-0063-2

**Published:** 2017-03-20

**Authors:** Damian Matak, Klaudia K. Brodaczewska, Monika Lipiec, Łukasz Szymanski, Cezary Szczylik, Anna M. Czarnecka

**Affiliations:** 1 Laboratory of Molecular Oncology, Department of Oncology, Military Institute of Medicine, Szaserow 128, 04-141 Warsaw, Poland; 2 School of Molecular Medicine, Medical University of Warsaw, Warsaw, Poland; 3 Faculty of Pharmacy with Laboratory Medicine Division, Medical University of Warsaw, Warsaw, Poland; 4 Institute of Genetics and Biotechnology, Faculty of Biology, University of Warsaw, Warsaw, Poland; 5 Department of Microwave Safety, Military Institute of Hygiene and Epidemiology, Warsaw, Poland

**Keywords:** RCC, Hypoxia, Hanging drop, Colony, Methylcellulose

## Abstract

Renal cell carcinoma (RCC) is the most lethal of the common urologic malignancies, comprising 3% of all human neoplasms, and the incidence of kidney cancer is rising annually. We need new approaches to target tumor cells that are resistant to current therapies and that give rise to recurrence and treatment failure. In this study, we focused on low oxygen tension and three-dimensional (3D) cell culture incorporation to develop a new RCC growth model. We used the hanging drop and colony formation methods, which are common in 3D culture, as well as a unique methylcellulose (MC) method. For the experiments, we used human primary RCC cell lines, metastatic RCC cell lines, human kidney cancer stem cells, and human healthy epithelial cells. In the hanging drop assay, we verified the potential of various cell lines to create solid aggregates in hypoxic and normoxic conditions. With the semi-soft agar method, we also determined the ability of various cell lines to create colonies under different oxygen conditions. Different cell behavior observed in the MC method versus the hanging drop and colony formation assays suggests that these three assays may be useful to test various cell properties. However, MC seems to be a particularly valuable alternative for 3D cell culture, as its higher efficiency of aggregate formation and serum independency are of interest in different areas of cancer biology.

## Introduction

Renal cell carcinoma (RCC) is the most lethal among the common urologic malignancies. RCC comprises 3% of all human neoplasms and comprises 90–95% of neoplasms arising from the kidney. According to the American Cancer Society, from 1975 to 2006, the incidence of kidney cancer rose 2% annually (Jemal et al. 2009). However, even after tumors are completely removed, RCC can easily recur locally or as metastatic disease (Chae et al. 2005). The mechanisms underlying disease dissemination and specific organ metastasis development are unknown (Bellmunt et al. 2011; Arai et al. 2008; Ami et al. 2005). Therefore, we need new approaches to target tumor cells that are resistant to current therapies and that give rise to recurrence and treatment failure (Bellmunt et al. 2011; Schmidinger et al. 2010). This goal cannot be reached if we continue to research cancer using two-dimensional (2D) hyperoxic monolayer models, which are far away from in vivo conditions.

Low oxygen tensions maintain undifferentiated states of embryonic, hematopoietic, mesenchymal, and neural stem cell phenotypes and also influence proliferation and cell-fate commitment. Recent evidence has identified a broader spectrum of stem cells influenced by low oxygen tension including some cancer stem cells: glioma stem cells, colon cancer stem cells, prostate cancer stem cells, and lung cancer stem cells (Heddleston et al. 2009; Anderson et al. 2011; Eliasz et al. 2010; Mohyeldin et al. 2011). Based on these data, it would be intriguing if kidney cell features are also regulated by oxygen tension, especially in the era of anti-angiogenic treatment of RCC (Bellmunt et al. 2011; Escudier et al. 2007; Dudek et al. 2009). Hypoxic microenvironments have been observed in solid tumors (Hockel and Vaupel 2001; Vaupel et al. 2001); this phenomenon is present when a tumor exceeds a certain size. Before that tumor size is reached, oxygen tension is slightly higher (Carreau et al. 2011); the pO_2_ value at the beginning of carcinogenesis is equal to the pO_2_ of a normal kidney (physioxia). We believe that the most important step of carcinogenesis is the very beginning of cancer development. Unfortunately, most researchers perform experiments under “normoxic/hyperoxic” cell conditions, where standard oxygen partial pressure is approximately 20%.

Due to the architecture of body tissues, three-dimensional (3D) microenvironments appear more favorable simulating in vivo conditions, since cell growth proceeds under more physiological conditions than in conventional 2D systems (Altmann et al. 2011; Smith et al. 2011). There are few studies supporting the impact of culture dimension on changes in gene expression profiles (Debeb et al. 2010). The creation of a third dimension for cell culture is more relevant to in vivo physiology and allows us to develop co-cultures for stem cell integration. In addition, 3D cell culture requires a multidisciplinary approach and expertise (Haycock 2011). The uses of 3D cell culture vary from clinical delivery to improved high-throughput drug screening systems. In cancer research, researchers constantly seek to improve relevant in vitro models for drug molecule discovery. Understanding cancer biology at the earliest stage of cancer development will allow us to propose new therapeutic targets. Therefore, within this study, we focused on low oxygen tension (hypoxia) and 3D cell culture incorporation to develop new RCC growth models.

## Materials and methods

### Cell lines

Human primary RCC cell lines (786-O, 769-P, CAKI-2, SMKT-R2, RCC6), a metastatic RCC cell line (ACHN), human kidney cancer stem cells (HKCSC), and human healthy kidney epithelial cells (ASE-5063) were used for experiments. The RCC lines 786-O, 769-P, CAKI-2, and ACHN were purchased from ATCC (Manassas, VA, USA). Human kidney cancer stem cells (HKCSC) were purchased from Celprogen (Torrance, CA, USA). The human kidney epithelial cell line (ASE-5063) was purchased from Applied StemCell, Inc. (Milpitas, CA, USA). RCC6 cells were kindly gifted by Prof. Salem Chouaib (INSERM, The Institut Gustave Roussy, Villejuif, France). The SMKT-R2 cell line was gifted by Prof. T. Tsukamoto and Dr. S. Tochizawa (School of Medicine, Sapporo Medical University, Sapporo, Japan). Cell lines were analyzed up to the 10th passage originally stocked in our laboratory.

### Cell culture maintenance

Cells were cultured in 75 cm^2^ cell culture flasks (Orange Scientific, Braine-l'Alleud, Belgium) in RPMI-1640/GlutaMax medium (Gibco, Grand Island, NY, USA), except for HKCSCs, which were maintained in Human Kidney Cancer Stem Cell Complete Growth Medium (Celprogen). RPMI-1640 was supplemented with 10% FBS (Hyclone, Logan, UT, USA, or Gibco) and treated with antibiotics (Penicillin–Streptomycin solution, Sigma, St. Louis, MO, USA) to a final concentration of 100 IU/mL penicillin and 100 μg/mL streptomycin. After thawing, cells were passaged at least once before using them in experiments. After reaching 80% confluence, cells were harvested by trypsynization (0.25% trypsin, 0.03% EDTA solution, Life Technologies, Carlsbad, CA, USA), counted in an automated cell counter (MOXI Z, Orflo Technologies, Ketchum, ID, USA), and used in the experiments described below. Cells were cultured per standard mammalian tissue culture protocols using sterile technique in normoxic (20% O_2_; 5% CO_2_; 37 °C), and hypoxic (1% O_2_; 5% CO_2_; 37 °C) incubators.

### Colony formation assay

The ability to create colonies was verified using the semi-soft agar method. Cells after the passage were collected as described and 2000 cells were seeded into a single well of a 96-well plate according to the Stem Cell Colony Formation Assay (Cell Biolabs, San Diego, CA, USA) protocol. Plates were placed in hypoxic and normoxic incubators. Cells were cultured for 14 days and photos of the colonies were taken using an inverted light microscope (Olympus, Tokyo, Japan).

### Hanging drop assay

Cells were collected after the passage as described and seeded at 500 cells per 15 µl drop of an appropriate medium on the inner side of a 100-mm dish lid. The lid was turned upside down and placed on top of a plate filled with 10 ml PBS, and placed into hypoxic and normoxic incubators. Cells were observed daily for 12 days to detect creation of aggregates. Photos were taken using an inverted light microscope (Olympus).

### 3D cell culture with methylcellulose

Cells after the passage were collected as described and used for 3D culture with methylcellulose (MC). Frozen methylcellulose containing medium (3% stock solution, R&D Systems, Minneapolis, MN, USA) was thawed at 4 °C. Working concentrations ranging from 0.01 to 1% of MC were prepared by dilution with RPMI with varying amounts of FBS (0 or 10% final concentration of serum) in sterile tubes. Due to the high viscosity of the medium, MC stock solution was handled with sterile syringes, while ≤1% MC-containing fluids were transferred with wide-mouth serological pipettes or tips (ex. 1000 µl tip). Cells were added to MC-containing media at a density of 10,000 cells/ml and mixed well. Air bubbles were allowed to escape and 100 µl (containing 1000 cells) were gently transferred to the bottom of a 96-well conical bottom (V) plate (Nunc, Roskilde, Denmark). To reduce evaporation, the edge rows of the plate were filled with sterile PBS to maintain proper concentration of MC throughout the experiment, as the MC might thicken upon water loss. Adding medium on top of MC medium is not advised as it will dilute methylcellulose, interfering with the experiment. Plates were each placed in normoxic and hypoxic incubators and cultured for 10 days. 3D structure formation was monitored using an inverted light microscope (Olympus).

## Results

### Hanging drop cell culture

**Fig. 1:**
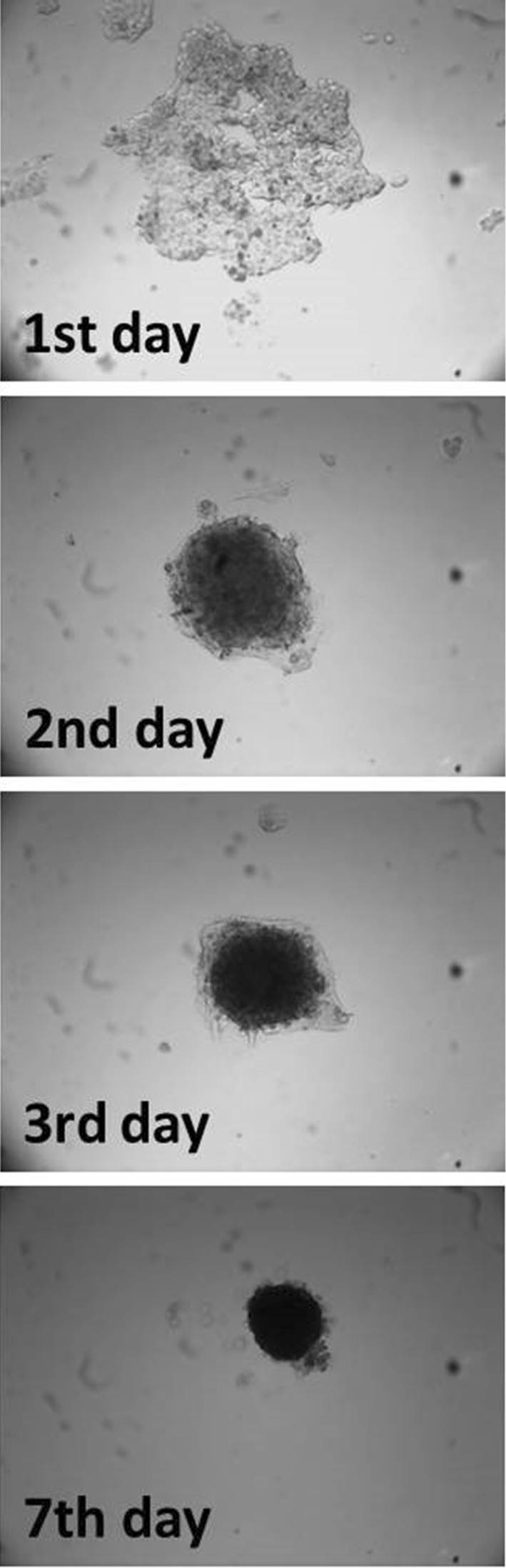
The process of compact aggregate creation. Changes in 786-O morphology under normoxic conditions in time—hanging drop assay. Magnification ×40

**Fig. 2:**
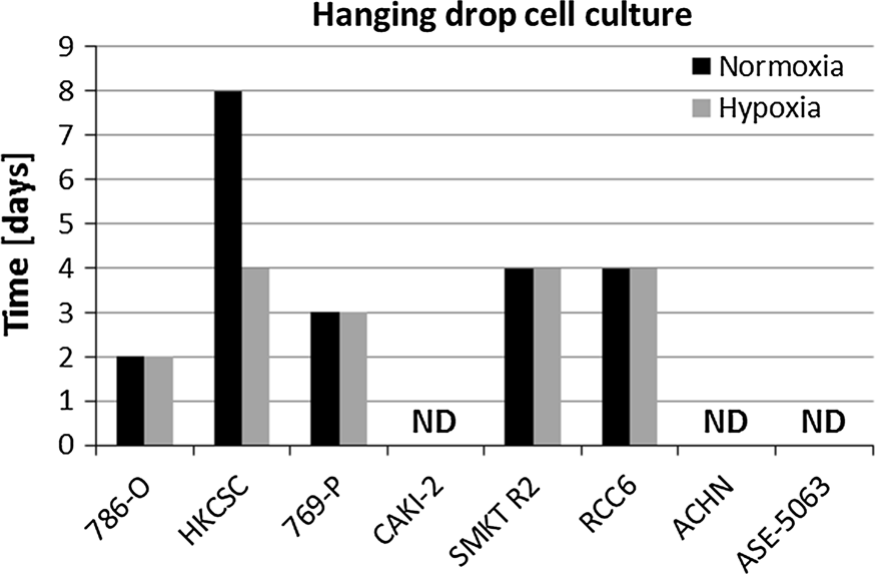
Differences in aggregation potential of various cell lines; cells with higher potential to aggregate showed reduced time. Caki-2, ACHN, and ASE-5063 were not able to create aggregate

**Fig. 3:**
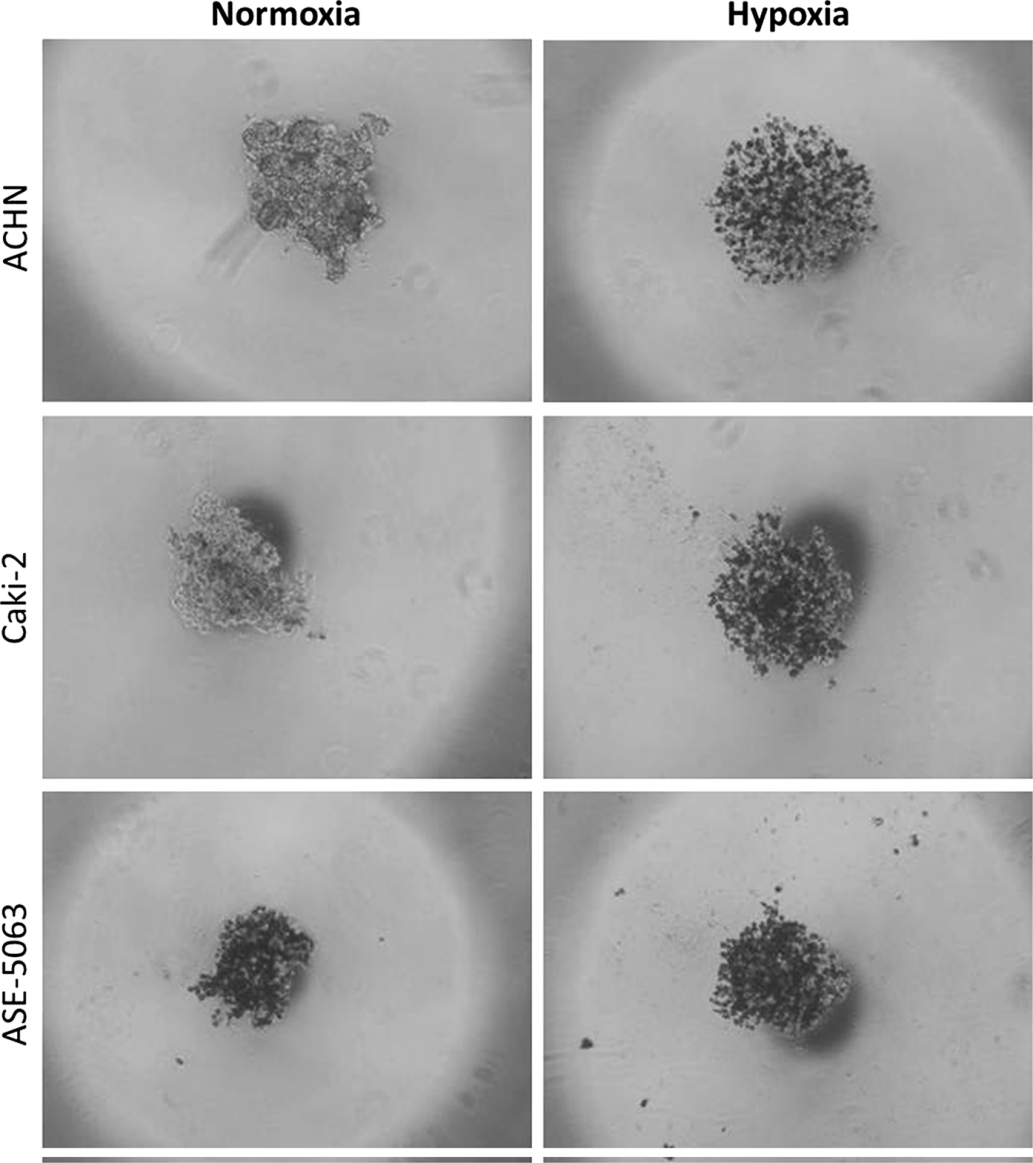
Morphology of ACHN and ASE-5063 cells in normoxia after the 12th day of hanging drop culture. Magnification ×40

Within the study, we verified the potential of various cell lines to create solid aggregates in hypoxic and normoxic conditions. Figure 1 presents an example of 786-O aggregate morphology changes over time under normoxic conditions; the time to create solid aggregates was recorded as the second day. Using this methodology, the time for solid aggregate creation of other cell lines was recorded (Fig. 2). Variations were observed in the time needed to create solid and homogenous aggregates in different cell lines, although no influence of oxygen on this phenomenon was observed in the analyzed RCC cell lines 786-O, 769-P, SMKT-R2 and RCC6. Of these, only RCC6 was isolated from a metastatic site; another metastatic RCC cell line (ACHN) was not able to create compact aggregates. HKCSC was the only cell line to demonstrate compact aggregation significantly faster in hypoxia than in normoxia, on the fourth and eighth days, respectively. Although they required different amounts of time to create solid aggregates, hypoxic and normoxic HKCSC aggregates had similar morphology. Cell lines derived from renal cell carcinoma primary tumor (CAKI-2), metastatic site (ACHN), and kidney epithelial cells (ASE-5063) could not create solid aggregates even after prolonged culture under hypoxic and normoxic conditions (Fig. 3). However, CAKI-2 and ACHN morphology in hypoxia was different than in normoxia. CAKI-2 and ACHN hypoxic cells were less compacted and more granular than normoxic ones. In contrast, oxygen had no influence on hanging drop cell morphology of kidney epithelial cells (ASE-5063); the aggregates were granular and heterogeneous.

### Colony formation assay

**Fig. 4:**
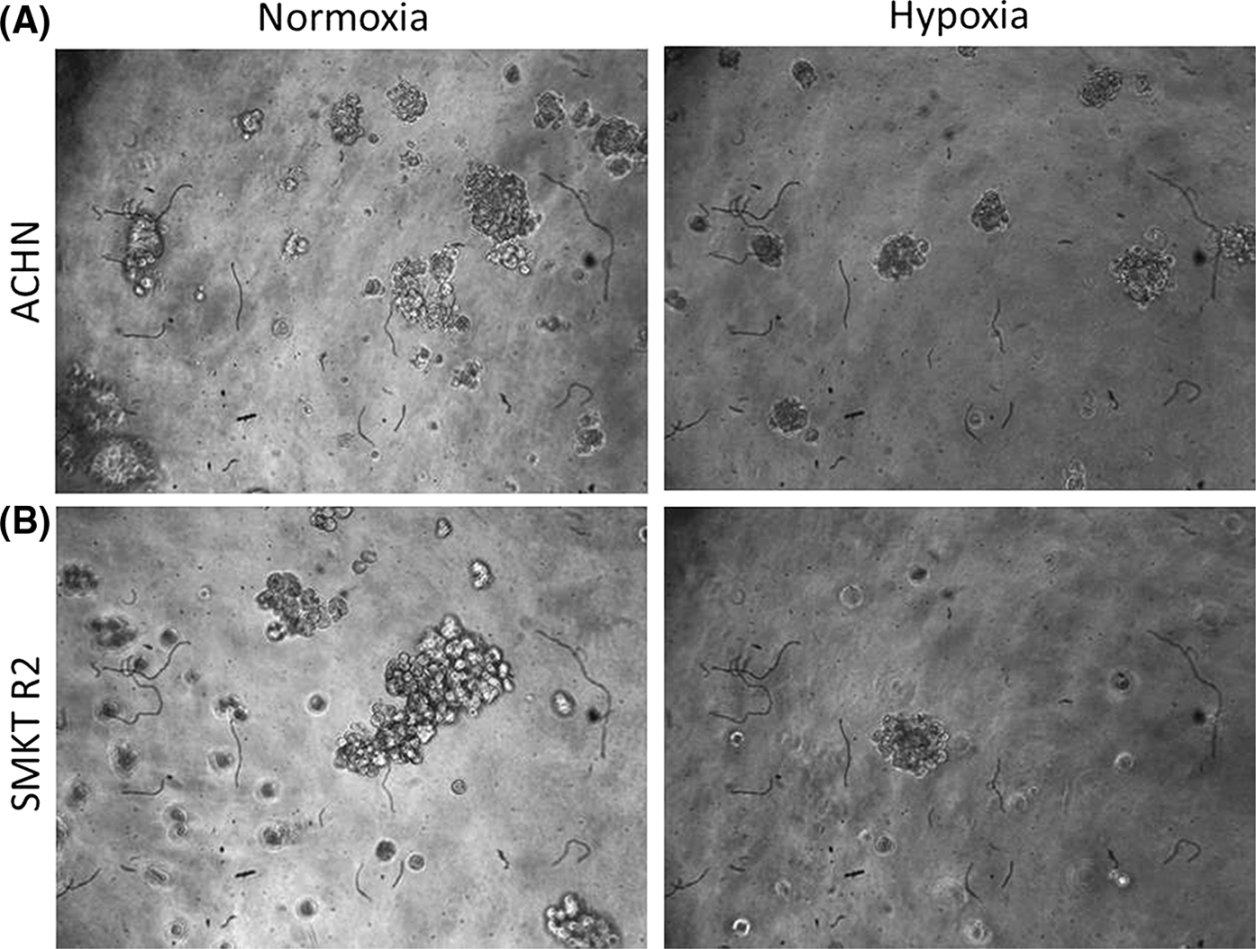
Semi-soft agar colonies of RCC cell lines in different oxygen partial pressures. Magnification ×40

The semi-soft agar method was used to determine the ability of various cell lines to create colonies under hypoxic or normoxic conditions. For this purpose, RCC cell lines derived from primary tumors (HKCSC, 786-O, SMKT R2, CAKI-2, 769-P, RCC6) and a metastatic site (ACHN) were analyzed. Only three cell lines generated colonies after 14 days of culture: HKCSC, SMKT R2, and ACHN. We found that hypoxia strongly reduces colony sizes in ACHN (Fig. 4a). A similar effect was observed in SMKT R2 (Fig. 4b), whereas no differences were noted in HKCSC (data not shown).

### Methylcellulose aggregation assay

Methylcellulose (MC), a cellulose derivative, is a polysaccharide that dissolves in aqueous solutions at temperatures below 50 °C, forming a viscous gel (Nasatto et al. 2015).

**Fig. 5:**
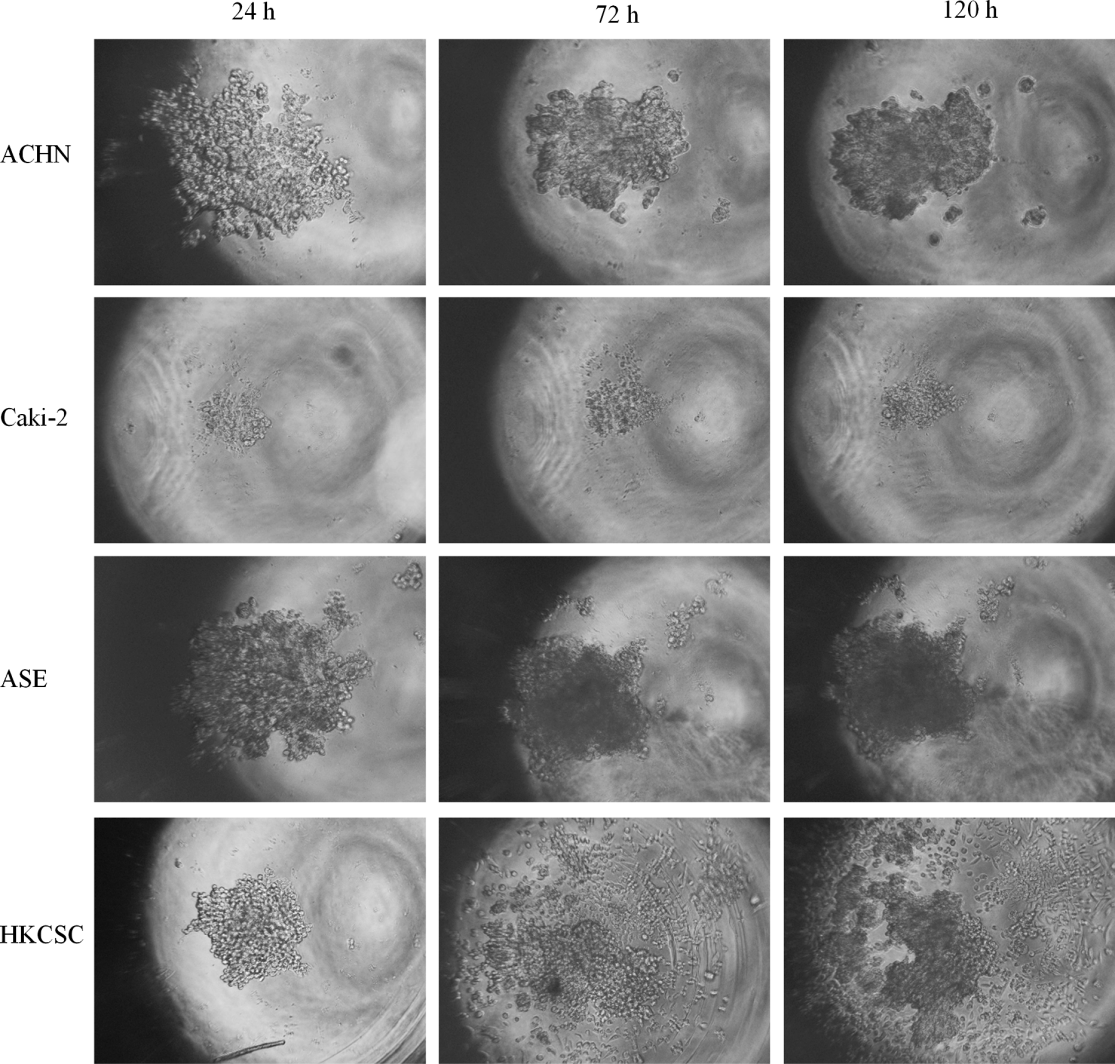
MC aggregates at different time lapses. Magnification ×40

In this analysis, both primary (ASE-5063 and HKCSC) and established metastatic (CAKI-2 and ACHN) cell lines were chosen as they have the same papillary origin as renal cell carcinomas (Looyenga et al. 2011; Kovacs et al. 1991; Furge et al. 2007; Pulkkanen et al. 2000). Cell lines formed aggregates at a higher rate with the methylcellulose method than with the hanging drop assay. Cells that did not create solid structures in the hanging drop assay could aggregate in MC. Cells accumulated near the bottom of the well as soon as 8 h after seeding and aggregates started to form around the first or second day of culture (Fig. 5). Spheroids continued to grow until the sixth day of culture, but were viable for as long as 10 days of cultivation (data not shown). However, formed structures differed from those observed in the hanging drop assay; they were less uniform and less compacted.

**Fig. 6:**
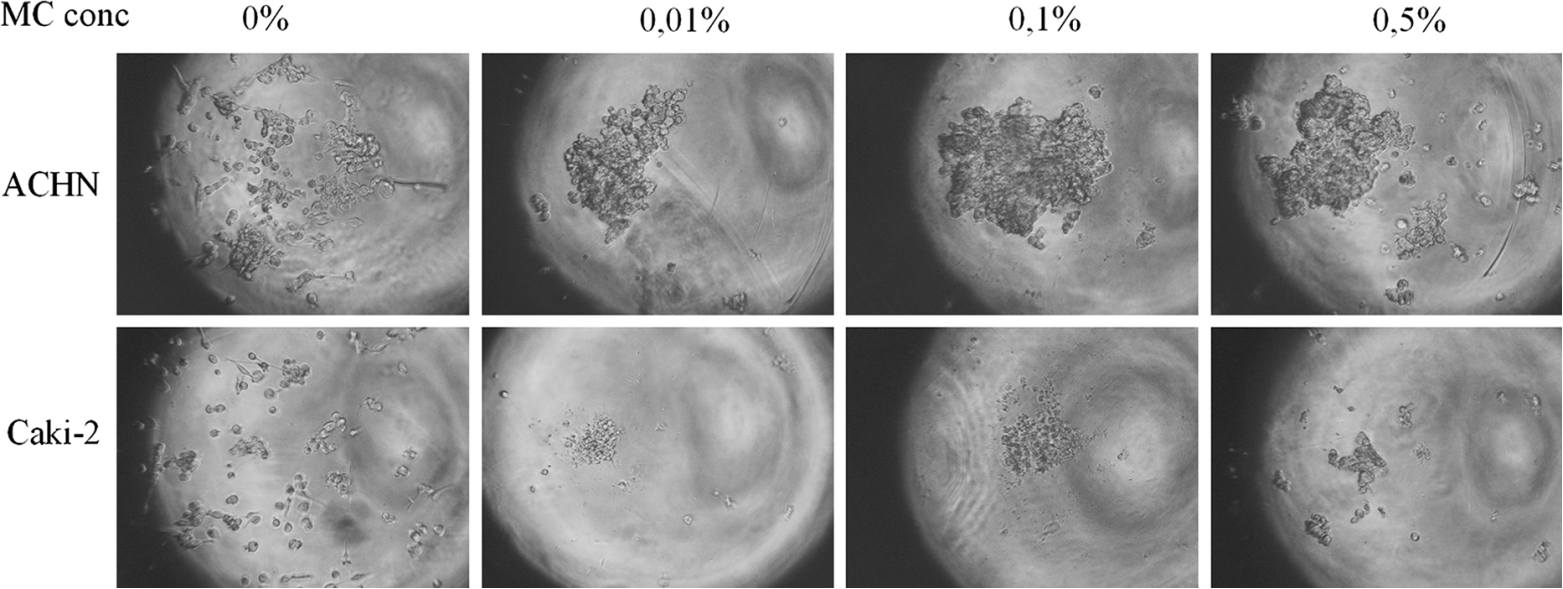
The influence of MC concentration on cellular aggregation. Magnification ×40

**Fig. 7:**
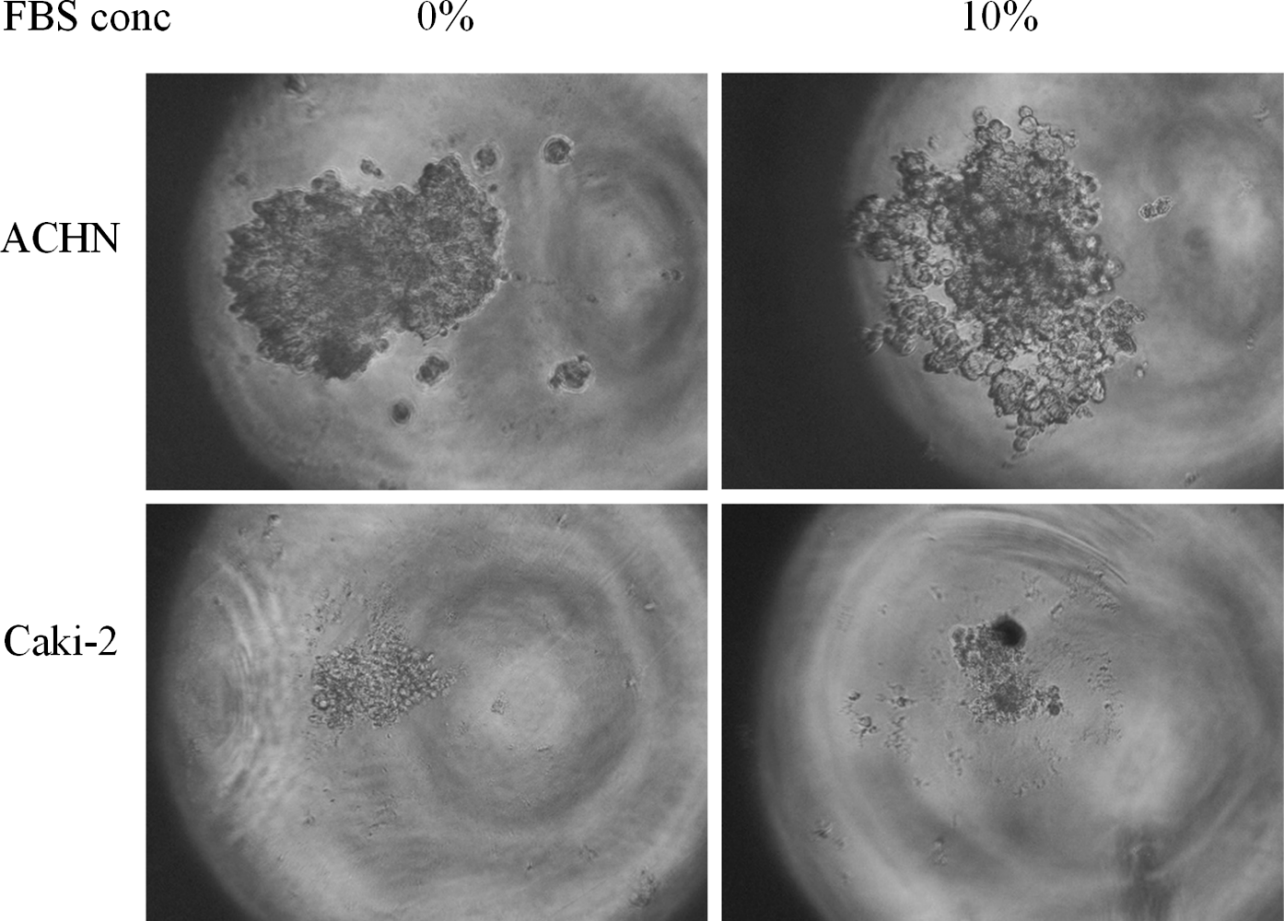
The influence of FBS concentration on cellular aggregation. Magnification ×40

Slightly different cell interactions may be active in forming 3D structures in MC as compared to in hanging drop. Different medium viscosity subjects cells to divergent forces (e.g., gravity) that may ultimately determine the morphology of formed structures. Structure morphology was also dependent on polymer and FBS concentration (Fig. 7). In the tested methylcellulose concentration range (0.01–1%), 0.05–0.25% MC seems to be an optimal starting point for most RCC cell lines (Fig. 6); however, it must be optimized by the end user. Higher concentration visibly resulted in formation of multiple structures, probably due to cell immobilization. In contrast, MC below 0.05% might be not enough to protect cells from adhering to the culture surface. Addition of FBS differentially affected spheroid morphology; ACHN cells compacted more in serum-free conditions, while CAKI-2 cells needed serum addition to form more rigid structures. Although the exact medium conditions need to be optimized for each cell line, the possibility of 3D structure formation in serum-free MC makes it a convenient tool for measuring the effect of different medium components and growth factors on the cells’ ability to aggregate into 3D structures (Fig. 7).

Interestingly, HKCSC cells that formed very compact structures in the hanging drop assay aggregated in MC within 24 h (in dedicated medium). However, during further culture, structures started to dislodge and cell outgrowth also took place as a monolayer (Fig. 5).

**Fig. 8:**
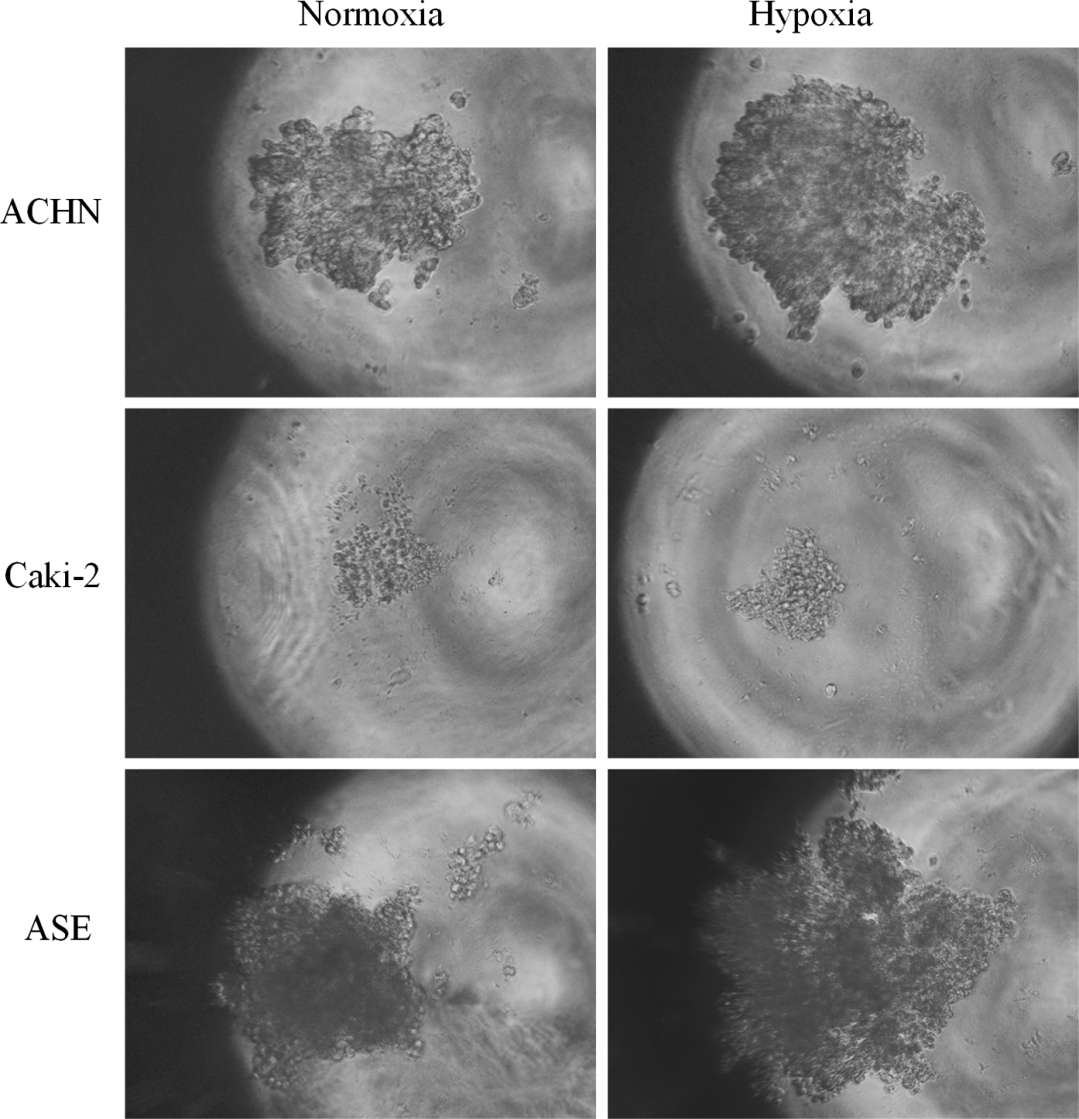
The influence of oxygen on cellular aggregation. Magnification ×40

As MC is a viscous polymer, the rate of gas diffusion and therefore oxygen equilibration may be different than in standard liquid media. We tested the effect of being cultured in reduced O_2_ pressure on cell aggregation and growth in low-concentration MC (0.01–1%). For all tested cell lines (CAKI-2, ACHN, HKCSC, and ASE-5063), culture under hypoxic conditions promoted growth of 3D structures; aggregates were bigger and more condensed, probably due to enhanced cell proliferation (Fig. 8). In other tested 3D culture methods, the effect of O_2_ concentration was line-dependent; ACHN and CAKI-2 cell aggregates in hanging drop were inhibited in hypoxia, while HKCSC compacted faster in low O_2_. This may suggest that the effect of hypoxia is different in standard and MC media. Both cell–cell interactions and O_2_ diffusion may be affected by MC viscosity.

### Growth summary

 See Table 1.

**Table 1:** Comparison of 3D structures formation in different culture conditions

Cell line	Hanging drop	Colony formation	MC assay
786-O	+	−	nt
HKCSCs	+	+	+
769-P	+	−	nt
CAKI-2	−	−	+
SMKT R2	+	+	nt
RCC6	+	−	nt
ACHN	−	+	+
ASE-5063	−	−	+

*nt* Not tested

“+” 3D structure formed

“−” No 3D structure formation

## Discussion

We analyzed various cell-culture based approaches to study the potential for 3D growth of renal cancer cells under hypoxic and normoxic conditions. Due to the architecture of body tissues, 3D microenvironments appear more favorable for simulating in vivo conditions than conventional 2D systems (Altmann et al. 2011; Smith et al. 2011). In the literature, there are few studies which support the impact of culture dimension on gene expression profile changes (Kenny et al. 2007; Edmondson et al. 2014). In our study, we used two common 3D culture methods, the hanging drop and colony formation assays, as well as one new method with methylcellulose and 96-well conical bottom plates.

### Hanging drop cell culture

The influence of the lack of oxygen on hanging drop culture in most RCC cell lines may be due to a dysfunctional mutation in *VHL*, which is important in RCC pathogenesis (Cowey and Rathmell 2009). Then again, the size of created aggregates (approximately 200 µm) may create an internal hypoxic region inside the aggregate. Strong hypoxic regions can be identified beyond 100 µm from the vessel wall (Tsai et al. 2003). Internal hypoxic environments may overlap with external hypoxia induced by the incubator, in which case no significant differences would be observed among their aggregate creation times and morphologies. On the other hand, solid aggregation time strongly depends on the cell line type, and observed differences could come from cell–cell interactions, reflecting different profiles of surface marker expression within cell lines (Foty 2011).

The hanging drop assay has been positively used to measure the capacity of cells to engage in cell–cell or cell-ECM (extracellular matrix) interactions, and to analyze cells in co-culture (two or more populations) (Foty 2011; Defresne et al. 2011; Kumar et al. 2014; Teng et al. 2014; Yip and Cho 2013). This method is very useful because it does not require any specialized equipment, and additional techniques such as fluorescent marker staining can easily be incorporated (Foty 2011). A new high-throughput method for hanging drop cell culture using specific well plates has recently been developed and studied (Tung et al. 2011; Hsiao et al. 2012; Burdett et al. 2010). Building on this method, a complementary spheroid transfer and imaging (TRIM) plate has been proposed (Cavnar et al. 2014). TRIM allows for incorporation of 3D system assays commonly used in biomedical research. This dynamic development illustrates the importance of the hanging drop culture method in bridging the gap between in vivo and in vitro physiology.

The relationship of oxygen partial pressure with the hanging drop assay has not been thoroughly studied in the literature. However, the hanging drop assay has been used to improve chondrogenic commitment of isolated adult human chondrocytes on cartilage formation (Martinez et al. 2008). Martinez et al. revealed highly organized 3D tissue-like structures using this method. The cultures were grown in different oxygen partial pressures. Cartilage from hypoxic cultures was bigger, with enhanced matrix deposition and higher quality cartilage formation compared to normoxic cultures. Differences in gene expression have also been observed in cells cultured in a 3D hanging drop system versus a standard monolayer culture. The gene expression profile from the 3D system was comparable to the profile from a pellet culture system. In summary, the combination of hypoxia and the hanging drop assay allows for easy generation of cartilage-like microstructures.

In a separate study, multicellular human mesenchymal stem cells (hMSC) were cultured in a hanging drop assay under hypoxic and normoxic conditions. ECM proteins, growth factors, and stemness were measured, and injectability, attachment, and integration within a tissue were verified. To conclude, Shearier et al. (2016) found that hypoxia enhances biomolecule production and stemness of hMSC, further showing that incorporation of low oxygen and hanging drop cell culture aptly reflects in vivo conditions. The researchers hypothesized that hypoxia may significantly improve hMSC potential by preventing cell differentiation. Sun et al. (2015) established another pivotal role of hypoxia in a hanging drop culture system. In mouse embryonic stem cells (ESC), overexpression of *HIF2alpha*, which is directly related to cell response to low oxygen tension, drives mouse ESCs to cardiomyocyte differentiation. This phenomenon may be very useful when applying ESC differentiation in translational medicine.

One of the most promising methods of hanging drop culture is the formation of pancreatic islet spheroids. Single islet cells were cultured under normoxic conditions using hanging drop cell culture. After few days, the morphology of aggregated islets was similar to intact islets. Moreover, treatment with various glucose concentrations showed that the glucose-mediated stimulation index was like that of intact islets (Kim et al. 2013). This confirms another useful application of the 3D cell culture system. In a previous study, Cavallari et al. (2007) also showed that the size of the aggregated islets strongly depends on the number of single cells. Moreover, cellular composition and architecture of reaggregated islets were comparable to intact islets.

The combination of hanging drop cell culture with a hypoxic environment has been limited to these studies and has proven to be a favorable cell culture condition for regenerative medicine. This may be because low oxygen partial pressure and 3D cell culture together are a good reflection of the in vivo microenvironment. However, our results did not show a significant influence of oxygen partial pressure on RCC aggregation potential. Nevertheless, we did observe some morphology differences. Our research is the first study of hanging drop cell carcinoma culture in hypoxia. We believe that further study using this method will allow us to develop an in vitro model to closely simulate in vivo conditions.

### Colony formation assay

Based on our data, the semi-soft agar method to mimic 3D cell growth is not very useful in an RCC model because of low colony formation efficiency. In hanging drop RCC culture, only two of the carcinoma cell lines failed to create homogenous and compact aggregates, but they still formed small aggregates with various cell densities. Even with a “negative” result, some 3D cell structures were observed. However, in the semi-soft agar method, lack of colonies meant the absence of any 3D cell structures. The hanging drop method thus seems more appropriate as an RCC 3D cell culture growth model. Although both models led to three-dimensional cell cultures, the created structures derived from other cellular properties.

The soft agar colony formation assay verifies the ability of cells to engage in anchorage-independent cell growth. Independent growth on a solid surface is a hallmark of carcinogenesis. The first example of this test was a clonogenic assay to evaluate cells’ abilities to form colonies (Puck et al. 1956). In that assay, cells were seeded in the presence of feeder cells or a conditioned medium. Over time, an easier method to verify colony formation was developed, which also allowed for detection of tumorigenic cell potential and the suppressive effects of many factors (Borowicz et al. 2014). Borowicz et al., proved that overexpression of two members of the WNT signaling pathway suppresses tumorigenic potential of murine lung carcinoma cells. In our study, we discovered that hypoxia inhibits colony formation in two out of seven RCC cell lines.

Importantly, the semi-soft agar colony formation assay can be useful in monitoring the effects of novel compounds on cell proliferation and migration (Horibata et al. 2015). The biggest advantage of the assay over a conventional monolayer culture is the close mimicry of the in vivo cellular environment. Horibata et al. identified the inhibitor potency of novel compounds (peptidylarginine deiminase enzyme inhibitor, BB-Cl-amidine) on human ductal carcinoma. Novel compounds had been previously identified as potential breast cancer biomarker and therapeutic targets (Horibata et al. 2012).

Many published studies use the soft-agar colony assay or describe assay protocols, and some companies even offer ready-to-use kits to analyze colony formation. Nevertheless, few studies examine both oxygen partial pressure and colony formation abilities. Zhou et al. (2011) used a colony formation assay to study a human glioblastoma cell line (U87) under hypoxic (2% oxygen) and normoxic conditions. In that study, in contrast to our results, hypoxia promoted colony formation of U87 and of a cell line with stem-like properties (U87-SC). Interestingly, U87-SC cells formed significantly more colonies than U87, indicating that U87-SC cells have a higher self-renewal capacity than U87 cells. Based on this result, SMKT-R2, ACHN, and HKCSC should have higher stem-like properties than other cell lines that could not generate colonies. Wang et al. (2011) also confirmed that hypoxia promotes colony formation and cancer cells with stronger stem-like properties have a higher colony formation ratio.

Other studies have demonstrated a relationship between oxygen partial pressure and cell growth. One study showed that clonogenicity of murine leukemia cells was better in 5% oxygen than in 20% oxygen (Fan et al. 1984). In addition to cancer cells, the clonogenicity of mouse embryo fibroblasts (MEFs) has been analyzed (DeYoung et al. 2008). Wild type MEF cells showed almost no influence of oxygen partial pressure on colony formation. Cloning efficiencies of human tumors derived from various sites including ovarian, lung, colon, breast, mesenchymal, and miscellaneous tissues have also been analyzed (Sridhar et al. 1983). Fresh patient tumor samples were plated in semi-soft agar and cultured in hypoxic and normoxic conditions. In most tumor types, hypoxia promotes cloning efficacy. However, in mesenchymal tumors, 5% oxygen decreased the number of colonies, similar to our RCC model results. In colon tumors, oxygen has no influence on colony formation. Another study revealed that cell colony formation abilities strongly depend on hypoxia-inducible factor 1-α (HIF-1α). The HIF-1α transcription factor is abundantly expressed in most human carcinomas and their metastases (Rohwer et al. 2008) and is the principal mediator of cellular adaptation to hypoxia. Cell lines with *HIF*-*1α*-knockdown displayed significantly reduced anchorage-independent proliferation (Rohwer et al. 2008). In a similar study, knockdown of *HIF*-*1α* by appropriate siRNA in a melanoma cell line decreased the colony formation potential of cells (Mills et al. 2009). Most RCCs are characterized by a dysfunctional mutation in the *VHL* gene (Cowey and Rathmell 2009), which leads to the constitutive expression of *HIF*-*1* (Hughson et al. 2003). This may indirectly explain why most of the RCC cell lines we tested, which do not generate colonies in normoxia, could not generate colonies in hypoxia.

### Methylcellulose aggregation assay

In biomedical research, methylcellulose solutions are used as an alternative to agar in colony forming assays, both for cancer cells and hematopoietic progenitors (Kubota et al. 1981; Neumann et al. 1984; Wognum et al. 2013). Here, we show that methylcellulose can be also used to test the aggregation abilities of cells. When used in lower concentrations (such as 0.05–0.25% as opposed to >0.8%) in a colony formation assay (Neumann et al. 1984) using conical bottom culture vessels, methylcellulose solution promotes cell aggregation and prevents cells from attaching to the culture surface even in serum-containing media. Such an approach may be an alternative to a hanging drop assay or to cultures in round bottom ultralow attachment plates.

Kojima et al. (2012) showed another interesting approach in using MC for cell aggregation: when cell suspension was injected into 3% MC, medium was absorbed leading to cell aggregation. Similar phenomena may be present when lower MC concentrations are used, as in our model. It may profoundly affect cell microenvironment; polymer swelling can manipulate the concentration of cell-secreted factors and therefore affect their auto- and paracrine action. Such MC properties could also affect the impact of hypoxia on cells; MC’s different gas capacity may influence the kinetics of hypoxia development in the media and inside cell aggregates. However, no data on such physical properties of MC could be found.

As MC remains fluid throughout the experiment, it can be readily washed off with media or buffers, leaving a spheroid available for other tests. Cell viability assays using Alamar Blue or MTT solutions can be performed to monitor cell number and health, as in other 3D models (Bonnier et al. 2015). Also, obtained structures can be moved and utilized in co-cultures and migration assays (Collet et al. 2016).

The conditions created by MC must be exactly characterized to verify which cell properties are used during aggregation under these conditions. Different cell behaviors in MC, hanging drop, and colony formation assays suggest that these three assays may be useful to test various cell properties. However, MC seems to be a valuable alternative particularly for hanging drop, as the MC method’s higher efficiency of aggregate formation and serum independency are relevant in different areas of cancer biology.

## Conclusions

The impact of hypoxia on the 3D development of kidney cancer is not clear. The results suggest that the cells’ response depends on their origins and variations. In hanging drop assays, hypoxia affects the morphology of aggregates in renal carcinoma cell lines, and reduces the time to create aggregates only in kidney cancer stem cell lines. In the soft agar method, the number of colonies created is lower in hypoxia. However, only two out of three cell lines could even grow colonies in hypoxia using this method. In methylcellulose, hypoxia promotes cell growth and aggregation. The differing behaviors of various cell lines in methylcellulose, hanging drop, and colony formation assays suggest that these three assays may be useful to test different cell properties. The choice of 3D cell culture method should take into consideration those various cell properties.

## Abbreviations

CFA: Colony formation assay
ECM: Extracellular matrix
ESC: Embryonic stem cell
HIF1α: Hypoxia-inducible factor 1-α
HIF2α: Hypoxia-inducible factor 2-α
hMSC: Human mesenchymal stem cell
MC: Methylcellulose
MEFs: Mouse embryo fibroblast
MTT: 3-(4,5-Dimethylthiazol-2-yl)-2,5-diphenyltetrazolium Bromide
RCC: Renal cell carcinoma
VHL: von Hippel–Lindau tumor suppressor
WNT: Wingless-type MMTV integration site family member
